# Localised prostate cancer treated with MRI-guided transurethral ultrasound ablation: phase I trial results

**DOI:** 10.1186/1470-7330-14-S1-S3

**Published:** 2014-10-09

**Authors:** MB Wolf, M Roethke, S Pahernik, B Hadaschik, T Kuru, IV Popeneciu, G Hatiboglu, J Chin, M Billia, J Relle, J Hafron, K Nandalur, M Burtnyk, HP Schlemmer

**Affiliations:** 1 Radiology, German Cancer Research Center (DKFZ), Heidelberg, Germany; 2 Urology, University Hospital, Heidelberg, Germany; 3 Urology, Western University (UWO), London Health Sciences Center, Victoria Hospital, London ON, Canada; 4 Urology and Radiology, Beaumont Health System, Royal Oak MI, USA; 5 Profound Medical Inc., Toronto ON, Canada

## Purpose

Purpose of this prospective, multi-institutional Phase I clinical study was to investigate whether MRI-guided transurethral ultrasound ablation (MR-TULSA), a novel minimally-invasive technology to treat organ-confined prostate cancer (PCa), is safe, feasible and effective. It employs directional plane-wave high-intensity ultrasound, which ablates prostate tissue using real-time thermometry with active temperature feedback control.

## Methods

Enrolled were 30 patients with biopsy-proven, low-risk prostate cancer (age ≥ 65y, T1c/T2a, PSA ≤ 10ng/ml, Gleason 6 (3+3)). Whole-gland prostate ablation was performed with MR-TULSA using the PAD-105 (Profound Medical Inc., Canada) and a 3T MRI (Siemens, Germany) in one single treatment session under general anaesthesia and 3D active MR-thermometry feedback control. Contrast-enhanced MRI (CE-MRI) immediately following the ablation and at 12 months confirmed thermal coagulation.

## Results

There were no intraoperative complications with normal micturition resuming after catheter removal. Median (range) treatment time and prostate volume were 36 (24–61) min and 44 (21– 95) ml, respectively. Maximum temperature during treatment depicted a continuous region of heating shaped accurately to the prostate within 0.1 ± 1.3 mm, with average over- and under- targeted volumes of 0.8 and 1.0 ml, respectively. Regions of acute cell kill on CE-MRI correlated well with treated volume on MR-thermometry. Successful treatment was further confirmed by a median PSA decrease from 5.35 to 0.70 ng/ml at 1 month (n=29), remaining stable to 0.65 ng/ml at 6 months (n=16).

## Conclusion

Phase I results show that MR-TULSA represents a minimally-invasive treatment option for safe, effective and accurate whole-gland thermal ablation of organ-confined prostate cancer.

